# Nasopharyngeal Meningococcal Carriage among Older Adults in Türkiye (MeninGOLD Study)

**DOI:** 10.3390/microorganisms11082095

**Published:** 2023-08-16

**Authors:** Pinar Yildiz, Melisa Sahin Tekin, Mucahit Kaya, Ener Cagri Dinleyici

**Affiliations:** 1 Department of Internal Medicine, Faculty of Medicine, Eskisehir Osmangazi University, Eskisehir 26040, Türkiye; 2 Diagen Biotechnology, Ankara 06070, Türkiye; 3 Department of Pediatrics, Faculty of Medicine, Eskisehir Osmangazi University, Eskisehir 26040, Türkiye

**Keywords:** *Neisseria meningitidis*, meningococci, invasive meningococcal infection, age, older adult, elderly, carriage

## Abstract

**Introduction**: While there is a significant amount of information about invasive meningococcal disease (IMD), meningococcal carriage, and meningococcal vaccines in children and adolescents, data in older adults are limited. Studies of meningococcal carriage and transmission modeling can be utilized to predict the spread of IMD and guide prevention and treatment strategies. Our study’s main objective was to assess the prevalece of *Neisseria meningitidis* (*Nm*) carriage, serogroup distribution, and associated risk factors among older adults in Türkiye. **Methods**: Nasopharyngeal samples were collected between December 2022 and January 2023 from a total of 329 older adults (65 years of age and above). The samples were tested via PCR for *Nm*, and a serogroup (A, B, C, Y, W, X, E, Z, H) analysis of the positive samples was performed. **Results:** In total, 329 adults over 65 years of age (150 females and 179 males; 69% were 65–75 years old and 31% were 75 years of age and older) were included in the study. *Nm* carriage was detected in 46 participants (13.9%), and the serogroup distribution was as follows: 2.4% MenY (*n* = 8), 1.8% MenB (*n* = 6), 0.2% MenW (*n* = 2), and 9.4% non-groupable (*n* = 31). Other serogroups were not detected. Between the meningococcal carriers and the non-carriers, there were no differences between previous vaccination histories (meningococcal, pneumococcal, influenza, and COVID-19), travel history for Hajj and/or Umrah, and the presence of chronic disease. Of the 16 cases positive for the serogroups Y, B, and W, 13 patients were between the ages of 65 and 74 and three patients were over 75 years old, and these three cases represented MenY. **Conclusion:** In our study, the percentage of meningococcal carriage was found to be 13.9%, the carriage rate for encapsulated strains was 4.8%, and the most common serogroup was MenY. Men Y was also the only serogroup detected in patients over 75 years of age. The MenY serogroup, which is one of the most important causes of IMD (especially in pneumonia cases) in people older than 65 years, was the most frequently carried serogroup in people over 65 years of age in our study. Adequate surveillance and/or a proper carriage study would help to define potential vaccination strategies for older adults.

## 1. Introduction

Invasive meningococcal disease (IMD) is a life-threatening disease caused by *Neisseria meningitidis* (*Nm*). A polysaccharide capsule is the main source of virulence; regarding the polysaccharide capsule, there are twelve *Nm* serogroups, and six serogroups (A, B, C, W, X, and Y) are commonly detected. Despite successful antibiotic and supportive therapies, approximately 10% of patients die, and survivors may have catastrophic outcomes such as amputation, neurologic and behavioral issues, and hearing loss [1]. The occurrence of IMD and the patterns of serogroup distribution reveal differences based on age group, geographical location, and the impact of meningococcal immunization programs [2]. Vaccination remains the most effective method of preventing IMD, and antibiotics are advised for post-exposure prophylaxis and therapy [1,2].

Although IMD is more prevalent in children less than five years of age, adolescents, and young adults, it has also been recorded in the geriatric population, perhaps as a result of known epidemiological changes in infectious diseases through time. The elderly have a reduced index of suspicion for IMD; atypical manifestations are far more common, and the disease has a higher case fatality rate (CFR). Social crowding, living with young children and adolescents, attending mass gatherings (like the Hajj or Umrah pilgrimages), residential nursing homes, and socioeconomic deprivation may all contribute to the spread of *Nm* and the development of IMD among the elderly [3]. Routine immunization with meningococcal vaccines is not currently available/recommended for older adults.

*Neisseria meningitidis* can be transmitted asymptomatically (in approximately 3–20% of the population) in the nasopharynx [1,2,3,4,5]. Most people become carriers several times over the course of their lives [5]. Despite the fact that meningococcal colonization is the initial step in the development of IMD, very little is understood about the risk factors that predict the transition from an asymptomatic to a clinically significant state [4,5,6]. The rates of meningococcal carriage show variability across different age groups and geographical regions [1,4,5,6,7]. The highest rates of carriage are found in adolescents and young adults [4]. In addition to age and geography, crowding conditions, exposure to cigarette smoke, previous respiratory infection, certain adolescent and young adult social behaviors, and antibiotic use have all been connected to meningococcal carriage [4,5,6,7,8,9].

The frequency of carriers for *Nm* and serogroups by age group are critical for determining disease burden, predicting outbreaks, and developing an effective immunization strategy [1,4,6]. Estimates of carriage prevalence across all ages are important for elucidating the epidemiology and transmission dynamics of meningococcal infection. Adults over the age of 65 are underrepresented in studies of IMD and meningococcal immunization, in contrast to younger age groups, and there is also a lack of meningococcal carriage data in the older population [3,10]. This prospective study aims to evaluate the prevalence of meningococcal carriage in individuals over 65 years of age in Türkiye to define circulating *Nm* carrier strains and to identify risk factors associated with meningococcal carriage among this age group.

## 2. Materials and Methods

### 2.1. Study Population

MeninGOLD is a prospective single-center study that was carried out in the Department of Internal Medicine, Eskisehir Osmangazi University Faculty of Medicine, between December 2022 and January 2023. The Clinical Research Ethics Committee at Eskisehir Osmangazi University approved the study (10 November 2022/32). In this trial, all procedures followed the guidelines set forth by the institutional and/or national research committee and the Declaration of Helsinki (1964) and its later amendments or other ethical standards. All participants provided written informed consent prior to the study and sampling. This study was financially supported by an Eskisehir Osmangazi University Research Grant (TCD-2023-2663).

We enrolled adults above 65 years of age who agreed to participate this study. Participants who had used antibiotics in the 8 weeks before the study were excluded. The participants were asked to complete a questionnaire. Age, gender, smoking status, presence of underlying diseases, travel history for Hajj and/or Umrah, and previous COVID-19 infections were recorded. Previous immunization histories for the pneumococcal vaccine (conjugated and/or polysaccharide), meningococcal vaccine, influenza vaccine, and COVID-19 vaccines, if available, were obtained.

### 2.2. Sampling

Cotton swabs (Copan Diagnostics, Carlsbad, CA, USA) were used to collect nasopharyngeal samples, which were then placed in an STGG medium (MC002; Diagen, Ankara, Türkiye). Charcoal Amies transport tubes were used to convey these samples to the lab. All sampling was carried out by the same member of the study team.

### 2.3. Laboratory Analysis

#### 2.3.1. Genomic DNA Extraction

DNA isolation, an evaluation of the presence of *Neisseria meningitidis*, and serogroup definition in all *Nm*-positive samples were performed. The swabs stored in the STGG medium were extracted using a DiaRex^®^ Brand Whole Blood Genomic DNA Extraction Kit (BLD-5295, Diagen, Ankara, Türkiye). Briefly, 200 µL of liquid sample was placed in a 1.5 mL microcentrifuge tube. Then, 250 µL of Lysis (LBD) solution was added. After a short vortex, 25 µL of Proteinase K (PKD) solution was added and incubated at 56 °C for 15 min. After incubation, 250 µL of absolute ethanol was added to the lysate, and all contents were homogenized via pipetting and transferred to the column. The column was centrifuged at 8000× *g* 1 min and transferred to a new tube. After washing the sample according to the kit protocol, 50 µL of elution (EBD) solution was added, and after 2 min of incubation, genomic DNA was obtained via centrifugation at 8000× *g* for 1 min. For long-term storage, the DNA samples gathered as a consequence of this study were kept at 80 °C. 

#### 2.3.2. Neisseria Meningitidis Screening 

The simultaneous detection of bacterial agents was carried out using a single-tube multiplex PCR analysis. This was achieved using a Meningococcus Multiplex Real-Time HD-FRT Kit (ECD-3101; Diagen, Ankara, Türkiye). The study was performed following the rules specified in the kit protocol. Briefly, 10 µL of master mix, 5 µL of oligo mix, and 5 µL of genomic DNA were used in a total volume of 20 µL. The positive and negative controls included in the kit were also included in the study, and the study was performed based on the TaqMan principle. The study was performed on a Bio-Rad CFX 96 (Bio Rad, Hercules, CA, USA), using 1 cycle at 95 °C for 5 min, followed by 40 cycles of 95 °C for 10 s, 59 °C for 30 s (read), and 72 °C for 5 s. Using sodC, ctrA and porA, a general screening was carried out. Samples found to be positive for *Nm* were recorded according to the result evaluation report provided in the kit.

#### 2.3.3. Serogroup Detection 

A (Orf-2), B (Sia D), C (Sia D), Y (Sia D), X (CtrA), W (Sia D), E (cseE), Z (cszC), and H (cshC) serogrouping was performed on the samples that tested positive for *Nm* in this investigation. This was achieved using a Meningococcal Serotyping (A/B/C/W/X/Y/E/Z/H) Real-Time PCR Kit (ECD-3102; Diagen, Ankara, Türkiye). The study was performed following the rules specified in the kit protocol. Briefly, 10 µL of master mix, 5 µL of oligo mix (A/B/C/W/X/Y/E/Z/H), and 5 µL of genomic DNA were used in a total volume of 20 µL. The positive and negative controls included in the kit were also included in the study, and the study was performed based on the TaqMan principle. The study was performed on a Bio-Rad CFX 96 (Bio Rad, Hercules, CA, USA), using 1 cycle at 95 °C for 5 min, followed by 40 cycles of 95 °C for 10 s, 59 °C for 30 s (read), and 72 °C for 5 s. Samples found to be positive for a *Nm* serotype were recorded according to the result evaluation report specified in the kit.

### 2.4. Statistical Analysis

A frequency analysis was conducted, and the chi-square test was used for comparisons. The statistical software SPSS for Windows, version 28.0 (Chicago, IL, USA), was used for all statistical analyses conducted in this study. Statistical significance was assumed when the *p*-value was less than 0.05. 

## 3. Results

This study involved the recruitment of 329 individuals aged 65 years and above and was conducted between December 2022 and January 2023. Nasopharyngeal samples were collected and subjected to PCR testing for the detection of Nm, and a subsequent analysis of serogroups (A, B, C, Y, W, X, E, Z, and H) was conducted using the positive samples. The study population comprised 150 females and 179 males. A total of 227 participants, accounting for 69% of the sample, fell into the age range of 65–75 years, while the remaining 102 participants, constituting 31% of the sample, were aged 75 years and older. Out of the total sample size of 329 participants, it was observed that 83.3% (*n* = 274) had at least one pre-existing chronic disease, whereas 44.3% (*n* = 146) had multiple underlying conditions. The percentages of chronic conditions are shown in Table 1. 

Regarding the participants’ immunization records, 129 adults had received pneumococcal vaccines (39.2%), 150 had received influenza vaccines (45.6%), and 315 had received at least one dose of a COVID-19 vaccine (95.7%). Ninety-eight (29.7%) of the participants had experienced a previous symptomatic COVID-19 infection. A travel history to Saudi Arabia for Hajj and/or Umrah was present in 68 (20.6%) of the participants, and 16 out of 68 of these adults had a travel history in the last 5 years. The number of participants who had received the conjugated meningococcal vaccine (Men ACWY vaccine) was 26 (7.9%). (Table 1).

Meningococcal carriage was observed in a total of 46 participants, accounting for 13.9% of the study population. The distribution of serogroups among the carriers was as follows: the prevalence of MenY was found to be 2.4% (*n* = 8), the prevalence of MenB was found to be 1.8% (*n* = 6), the prevalence of MenW was found to be 0.2% (*n* = 2), and non-groupable strains accounted for 9.4% (*n* = 31). The presence of serogroups A, X, E, Z, and H was not observed. In the assessment of groupable serogroups, specifically omitting non-groupable isolates, a total of 16 out of 329 samples were identified (4.8%). The distribution of serogroups within this subset was as follows: eight samples were classified as MenY (representing 50% of all groupable serogroups), six samples were classified as MenB, and two samples were classified as MenW. 

In 227 participants who were 65–75 years of age, the percentage of meningococcal carriage was 5.7% (34/227), and the distribution of serogroups was as follows: Men B (*n* = 6), Men W (*n* = 2), and Men Y (*n* = 5). In the 102 participants who were 75 years of age and older, the meningococcal carriage rate was 2.9% (3/102), and all *Nm* were Men Y (Table 1). 

There was also no difference for the presence of a chronic underlying condition with respect to carrier status. Between the meningococcal carriers and non-carriers, there were no differences between previous vaccination histories (meningococcal, pneumococcal, influenza, and COVID-19), history of travel for Hajj and/or Umrah, or the presence of chronic disease. Out of the 16 individuals who were identified as carriers of meningococci, two of them (one carrying Men Y and one carrying MenW) had previously received meningococcal vaccines. Specifically, both individuals had been administered the polysaccharide meningococcal ACWY vaccine more than five years ago, prior to their journey to Saudi Arabia for the Hajj pilgrimage. 

## 4. Discussion

In this study, the overall meningococcal carriage rate was 13.9%, and the groupable meningococcal carriage rate was 4.8% among adults over 65 years old. The groupable meningococcal carriage rate was 5.7% in the group of participants 65–75 years old and 2.9% in the participants 75 years of age and older. The majority of meningococcal studies have been performed in children, adolescents, and young adults. Carrier data in the elderly are scarce in the scientific literature [3,10]. Christensen et al. [4] found that age was the most significant factor in determining the prevalence of meningococcal carriage in their systematic review. According to their model, carriage was predicted to be low in infants and toddlers, increase steadily until it reached a peak in 19-year-olds, and then decrease again in the middle of life and old age. Drayß and coworkers [10] reported the first-ever rates of meningococcal carriage in otherwise healthy adults aged 65 and older. A multi-center cross-sectional investigation of 677 asymptomatic German people aged 65 and older examined the carriage of *Neisseria meningitidis*, *Haemophilus influenzae*, *Streptococcus pneumoniae*, *Group A Streptococcus*, and *Staphylococcus aureus*. The rate of meningococcal carriage was 0.3 percent. Both carriers, aged 76 and 73, were female community-dwelling residents who had never come into contact with preschool-aged children [10]. Although the carriage rate was lower in their study than in ours, that research was conducted in 2012–2013. In meningococcal carriage studies, the frequency of carriage may vary. It may vary according to the country in which the study is conducted (even according to regions within the same country), the age and characteristics of the cases included in the study group, the time period of the study, the method of sampling, and the microbiological and molecular methods used in the study. The routine and widespread use of meningococcal vaccines in children and adolescents may also affect the seroepidemiology of carriage. The global population’s advancing age has contributed to the escalation of infections present in individuals with various risk factors, thus resulting in an increase in mortality rates associated with sepsis. Hence, it is crucial to ascertain the prevalence of carrier rates for microorganisms like meningococcus in the broader community as it supports the strategic planning of preventive interventions.

Previous research findings indicate that older adults are more susceptible to IMD, its associated sequelae, health-care costs, and mortality rates [3,11]. It has been observed that elderly persons experience atypical symptoms of IMD, making diagnosis challenging and allowing the condition to develop untreated. The highest case fatality rate is seen in these age groups, and the frequency of IMD cases in the elderly has been increasing over time [3,11]. In the present study, MenY was the most common serogroup for carriage, followed by MenB and MenW (when excluding non-groupable isolates). It has been previously shown that the serogroups B, Y, and W are more common for IMD among the elderly. Guedes et al. [3]. found that the serogroups W and Y accounted for the majority of IMD cases in elderly patients, despite the fact that they are not the most common strains worldwide. Our research found that among the elderly, these three serogroups (Men Y, B, and W) were the most common carriers. Meningococcal disease often manifests itself in the form of pneumonia in elderly people and is most commonly caused by the serogroups Men Y, W, and B [3,12]. Men Y is the leading cause of meningococcal pneumonia in persons over the age of 65 [12]. In our study, the most common serogroup among those aged 65 and older was Men Y, with a prevalence of 2.4%. Men Y also represented the only serogroup found in participants aged 75 and older.

Understanding the epidemiology and transmission dynamics of meningococcal infection requires knowledge of the regional and temporal variations in the serogroup distribution of IMD and *Nm* carriage [1]. Meningococcal disease seroepidemiology in Türkiye is dynamic and slightly different from in Europe and the United States [13]. According to surveillance data, *Nm* is the most prevalent bacterium responsible for pediatric bacterial meningitis, and MenB was the most common strain in Türkiye between 2005 and 2018, followed by MenW, MenA, and MenY [14]. Men C has not been reported from IMD surveillance studies or from carriage studies in Türkiye [13,14,15,16]. There are no meningococcal vaccines in the routine childhood immunization program. Military personnel and pilgrims to Mecca and Medina are required to get the MenACWY conjugated vaccine. The MenACWY conjugated vaccine and 4CMenB vaccines are both available through private practitioners. Meningococcal carriage rates among adolescents and young adults in Türkiye ranged from 6.3% to 7.5%, with Men W, B, X, A, and Y being the most common serogroups [17,18]. Serogroup distributions for IMD among the elderly in Türkiye, as well as carriage rates among this age group, remain unknown. Similar to the serogroups circulating for IMD and carriage among children and adolescents, we found that these three (Men Y, B, and W) were the most common carriage strains among the elderly in our study. Among adults and the elderly population, meningococcal vaccination is only suggested for patients who are members of various risk groups, including those participating in the Hajj or Umrah pilgrimages [11,17]. After serogroup B, serogroup W is the most frequent meningococcal serogroup in Türkiye; an epidemic was linked to Hajj visitors and their close connections in 2005 [13,14]. In Türkiye in 2018, routine immunization was initiated for Hajj pilgrims, who received a meningococcal conjugate ACWY vaccine before they left for Saudi Arabia [18]. In the present study, 26% of participants had previously traveled to Saudi Arabia for Hajj and/or Umrah; 16 of these 68 people had done so during the previous five years. The conjugated meningococcal ACWY vaccination coverage among the participants was 7.9%. Among the sixteen groupable meningococcal carriers, two meningococcal carriers (Men Y and MenW) had received polysaccharide meningococcal ACWY vaccines at least 5 years before traveling to Saudi Arabia for the Hajj. Tezer and coworkers [18] planned to evaluate whether vaccinating with the MenACWY conjugated vaccine would reduce the number of pilgrims who brought *Nm* back to Türkiye. At the time of departure, 3.9% of pilgrims were found to be carrying meningococcal bacteria; all positive samples were serogroup B, and 0.4% of pilgrims who were not carriers before their trip tested positive for serogroup B upon their return to Türkiye [18].

Certain strains of meningococci are non-groupable and do not express capsules [19]. The present study observed a meningococcal carriage rate of 9.4% for non-groupable strains. Although non-groupable or unencapsulated meningococci seldom result in invasive disease, they are frequently associated with meningococcal carriage [9,19]. The findings of a cross-sectional study conducted among Norwegian adolescents revealed that a majority of the carriage isolates observed were identified as capsule-null, accounting for 40.1% of the total [9]. A study conducted by Tekin et al. [14] investigated the prevalence of meningococcal carriage among adolescents and young adults in Türkiye, revealing an overall carriage rate of 6.3%, with 14.4% of the isolates categorized as non-groupable.

Patients with some underlying conditions have a higher risk of IMD, although those people only make up a small percentage of all IMD cases [1,2,8]. The vast majority of IMD instances occur in people who do not have any of the risk factors previously mentioned. Meningococcal pneumonia in the elderly is often accompanied by underlying respiratory comorbidities and is associated with common medical co-morbidities that are more common in older people (such as diabetes mellitus, chronic pulmonary and renal disease, and autoimmune disease) [12]. Our research found no significant difference in immunization status (pneumococcal, influenza, or COVID-19) between meningococcal carriers and non-carriers.

*Streptococcus pneumoniae*, influenza, and, more recently, COVID-19 infections, are leading causes of death and disability among the elderly. Vaccination against these diseases is a common practice for the elderly and is an integral part of the public health strategy for older adults [20,21]. Our group had a 39.2% uptake of pneumococcal vaccinations, a 45.6% uptake of influenza vaccinations, and a 95.7% uptake of at least one dose of a COVID-19 vaccination, according to the immunization data. Our institution may have a higher-than-average immunization rate for pneumococcal and influenza vaccines because of the type of patients we treat. In our study, between the meningococcal carriers and non-carriers, there was no difference between previous vaccination histories for pneumococcal, influenza, and COVID-19 vaccines.

Furthermore, this research was conducted after the COVID-19 epidemic. During the first year of the pandemic, IMD cases appeared to be declining in multiple countries, likely due to stringent infection control and lockdown measures minimizing close contacts and limiting social gatherings that would normally facilitate meningococcal transmission [22]. Some countries observed an increase in IMD cases after the second year of the pandemic [23]. Our research is the first to examine the prevalence of carriage in older adults. The majority of participants (95.7%) had at least one dose of the COVID-19 vaccine, and over 30% had experienced symptoms of a previous COVID-19 illness, according to immunization data. There is no correlation between meningococcal carriage and prior infection with COVID-19.

This study had some limitations. It is challenging to extend these results to other geographical areas because they are based on a single-center study. Carriage detection may have been affected by how the *Nm* samples were handled and the techniques utilized in the laboratory [5]. In this study, we performed a PCR for the detection. Primers must be carefully chosen when employing a PCR for detection and genogrouping to prevent underestimating carriage [5]. Primers for target genes, including ctrA, sodC, and porA, were employed in our study. While we did not detect all serogroups among the elderly population, in this study, we evaluated the serogroups A, B, C, Y, W, X, E, Z, and H to cover all potential carriage serogroups. Some *Nm* isolates show resistance to both ciprofloxacin and beta-lactam antibiotics, particularly in MenY in the United States, and this is a global concern [23]. The antimicrobial resistance to ciprofloxacin of MenY isolates from IMD cases and asymptomatic carriers is an important issue for chemoprophylaxis. In our study, we did not assess antibiotic resistance patterns. In our study, adults over 65 years of age who were admitted to the Internal Medicine outpatient clinic for routine control or other complaints, who did not have acute infection, and who had not used antibiotics in the last 8 weeks were included. an evaluation of carriage in elder care homes, where carriage may be common, or in other populations who do not apply to health care facilities, will provide clearer information about meningococcal carriage in the general population.

## 5. Conclusions

In our study, the meningococcal carriage rate of encapsulated strains was 4.8%, and the most common serogroup was MenY. The MenY serogroup, which is one of the most important causes of IMD (especially in pneumonia cases) in people older than 65 years of age, was the most frequently carried serogroup in people over 65 years of age in our study. Although the incidence of this disease is reduced in vaccinated children and adolescents, with the application of available meningococcal vaccines, there are no data as to whether these vaccines provide herd immunity for the older population. Epidemiological alterations for IMD among older persons have also brought to light the necessity for additional research and the expansion of active monitoring systems in order to appropriately characterize the shifting epidemiology of IMD. Carrier data in the elderly are scarce in the scientific literature; consequently, the evaluation of carriage rates will play a significant part in determining the sero-epidemiology of IMD and further potential immunization strategies. In the event that vaccination efforts for older persons are required, adequate surveillance and/or an appropriate carriage study would be helpful in defining those prospective strategies.

## Figures and Tables

**Table 1:** Age distribution, gender, and immunization status of the study group and a comparison regarding meningococcal carriage and serogroup status.

	Groupable *Nm* Carriage				Total *Nm* Carriage (*n* = 46)	Non-Carriers (*n* = 283)	Total (*n* = 329)
	Serogroup Y (*n* = 8)	Serogroup B (*n* = 6)	Serogroup W (*n* = 2)	Total (*n* = 16)			
**65–74 years**	5 (62.5%)	6 (100%)	2 (100%)	13 (81.3%)	34 (73.9%)	193 (68.2%)	227 (69.0%)
**>75 years**	3 (37.5)	0 (0%)	0 (0%)	3 (18.7%)	12 (26.1%)	90 (31.8%)	102 (31.0%)
**Male/female**	3/5	2/4	2/0	7/9	16/30	134/149	150/179
**Pneumococcal vaccines ^a^**	5 (62.5%)	1 (16.6%)	1 (50%)	7 (43.7%)	18 (39.1%)	111 (39.2%)	129 (39.2%)
**Influenza vaccine ^b^**	3 (37.5%)	2 (33.3%)	0 (0%)	5 (31.2%)	17 (36.9%)	133 (46.9%)	150 (45.6%)
**COVID-19 vaccine ^c^**	8 (100%)	3 (50%)	1 (50%)	12 (75%)	44 (95.6%)	274 (96.8%)	318 (96.6%)
**Previous COVID-19 infection**	2 (25%)	4 (66.6%)	0 (0%)	6 (37.5)	14 (30.4%)	84 (29.6)	98 (29.7%)
**Hajj/Umrah visit**	2 (25%)	1 (16.6%)	0 (0%)	3 (18.7%)	15 (32.6%)	53 (18.7%)	68 (20.6%)
**Last 5 years**	0 (0%)	1 (16.6%)	0 (0%)	1 (6.2%)	4 (8.6%)	9 (3.1%)	13 (3.9%)
**Meningococcal vaccines ^d^**	1 (12.5)	0 (0%)	1 (50%)	2 (12.5%)	4 (8.6%)	22 (7.7%)	26 (7.9%)
**Conjugated vaccine (MenACWY)**	0 (0%)	0 (0%)	0 (0%)	0 (0%)	2 (4.3%)	11 (3.8%)	13 (3.9%)
**Presence of chronic condition**	6 (75%)	6 (100%)	1 (50%)	13 (82.3%)	37 (80.5%)	237 (83.8%)	274 (83.3%)
**Hypertension**	4 (50%)	3 (50%)	0 (0%)	7 (43.7%)	26 (56.5%)	159 (56.1%)	185 (56.2%)
**Diabetes Mellitus**	2 (25%)	3 (50%)	1 (50%)	5 (31.2%)	16 (34.7%)	96 (33.9%)	112 (34.0%)
**Coronary artery disease**	0 (0%)	2 (33.3%)	0 (0%)	2 (12.5%)	6 (13.0%)	38 (13.4%)	44 (13.3%)
**COPD/Asthma**	0 (0%)	0 (0%)	0 (0%0	0 (0%)	1 (2.1%)	12 (4.2%)	13 (3.9%)
**Malignancy**	0 (0%)	0 (0%)	0 (0%)	0 (0%)	1 (2.1%)	13 (4.5%)	14 (4.2%)
**Others**	1 (12.5%)	4 (66.6%)	0 (0%)	5 (31.2%)	10 (21.7%)	74 (2.6%)	84 (25.5%)
**More than one chronic condition**	1 (12.5%)	6 (100%)	0 (0%)	10 (62.5)	17 (36.9%)	129 (45.5%)	146 (44.3%)

^a^: received 13-valent conjugated vaccine alone or with 23-valent polysaccharide pneumococcal vaccine; ^b^: trivalent or quadrivalent influenza vaccines; ^c^: at least 2 doses of COVID-19 vaccine (inactivated COVID-19 vaccine and/or COVID-19 mRNA vaccine); ^d^: polysaccharide MenACWY vaccine or conjugated MenACWY vaccine.

## Data Availability

The data are available from the corresponding author upon reasonable request.
